# Emergence of cationic polyamine dendrimersomes: design, stimuli sensitivity and potential biomedical applications

**DOI:** 10.1039/d1na00536g

**Published:** 2021-09-01

**Authors:** Partha Laskar, Christine Dufès

**Affiliations:** Department of Immunology and Microbiology, School of Medicine, University of Texas Rio Grande Valley McAllen TX 78504 USA partha.laskar@utrgv.edu; Strathclyde Institute of Pharmacy and Biomedical Sciences, University of Strathclyde 161 Cathedral Street Glasgow G4 0RE UK C.Dufes@strath.ac.uk

## Abstract

For decades, self-assembled lipid vesicles have been widely used in clinics as nanoscale delivery systems for various biomedical applications, including treatment of various diseases. Due to their core–shell architecture and versatile nature, they have been successfully used as carriers for the delivery of a wide range of therapeutic cargos, including drugs and nucleic acids, in cancer treatment. Recently, surface-modified polyamine dendrimer-based vesicles, or dendrimersomes, have emerged as promising alternatives to lipid vesicles for various biomedical applications, due to their ease of synthesis, non-immunogenicity, stability in circulation and lower size polydispersity. This mini-review provides an overview of the recent advances resulting from the use of biomimetic hydrophobically-modified polyamine-based dendrimersomes towards biomedical applications, focusing mainly on the two most widely used polyamine dendrimers, namely polyamidoamine (PAMAM) and poly(propylene imine) (PPI) dendrimers.

## Abstract 2

Emergence of surface-modified polyamine dendrimer-based vesicles (or dendrimersomes) as promising alternatives to lipid vesicles for biomedical applications.

## Graphical abstract

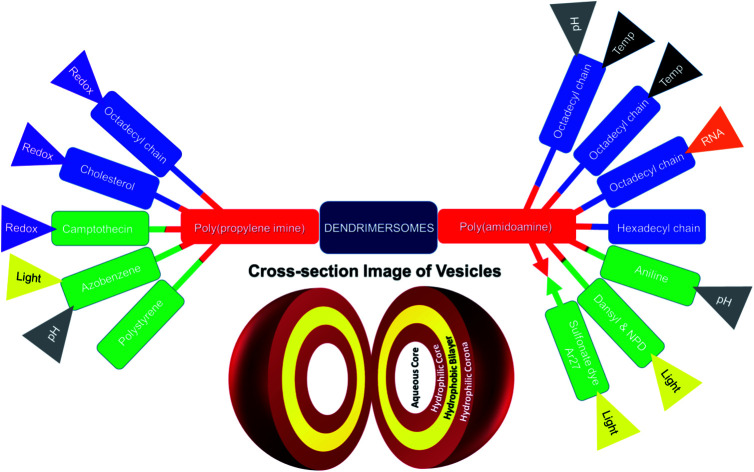

## 1. Introduction

Dendrimers are emerging as potential non-viral vectors for efficiently delivering drugs and nucleic acids to the brain and cancer cells. They are polymeric, nanometer-sized synthetic molecules with perfectly branched multiple monomers that emerge radially from a central core similar to a tree (*dendron* in Greek).^1–4^ Dendrimers consist of three main architectural components: a reactant core molecule, which is considered the origin of the dendrimer, highly branched polymeric layers (or generations) that bind to the core in a specific way to form a uniformly branched spherical macromolecule, and a multivalent periphery.^5,6^ Their modifiable surface functionalities, low polydispersity and available internal cavities make them particularly attractive as delivery systems for drug and gene delivery applications.^1,2,4–8^ Recently, the ability of surface-modified amphiphilic polyamine dendrimers to form self-assembled nanostructures in water was the object of much scrutiny. These amphiphilic dendrimers were synthesized by conjugating various hydrophobic groups (*i.e.* aromatic, long hydrophobic, fluorinated chains) to the hydrophilic dendrimers.^5,9–13^ The polyamine dendrimer-based vesicles, or dendrimersomes, resulting from their self-assembly, have emerged as promising delivery systems for various biomedical applications.

The self-assembly of amphiphilic molecules in water is a natural phenomenon resulting in various ordered thermodynamically stable nano or microstructures, which is mostly driven by various forces, such as hydrophobic interactions, hydrogen bonding and metal–ligand interactions.^14–17^ Hydrophobic interactions are important factors causing the self-assembly of amphiphilic molecules in aqueous solution, which actually led to the rational design and synthesis of various low- and high-molecular weight amphiphiles with various hydrophobes towards nano or micro assemblies in the last decades.^18–21^ Despite many potential applications, most artificial nanostructures constituted of low- and high-molecular weight amphiphiles showed inherent drawbacks due to their *in vivo* instability and inefficient delivery of cargos, which led this field evolving to develop enhanced nano-carriers for biomedical applications, especially for the delivery of therapeutic agents in cancer therapy.

Among various self-assembled nanostructures, vesicles, three-dimensional entities with an aqueous lumen separated from the outside aqueous medium by one or more hydrophobic bilayer, are widely studied delivery systems as they resemble the structure of naturally occurring cells, viral capsids or the recently developed exosomes.^22–26^ They are advantageous over other delivery systems for biomedical applications as they can carry hydrophobic as well as hydrophilic cargos to the target site of action. Several synthetic vesicles such as liposomes (lipid-based vesicles), polymersomes (polymer-based vesicles) and dendrimersomes (dendrimer-based vesicles) can mimic natural carriers and are currently under investigation for various biomedical investigations, such as therapy, diagnostic, analytical science and synthetic biology.

**Fig. 1:**
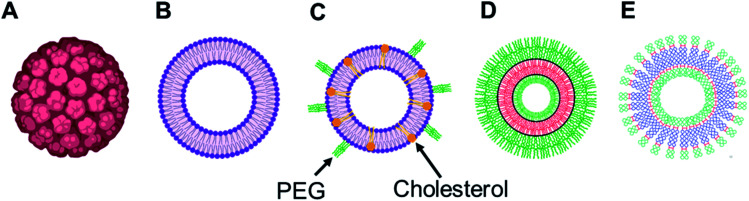
Representation of various vesicular structures: (A) viral capsid, (B) liposome (or lipid-based vesicle), (C) stealth liposome (with additives such as cholesterol and PEG), (D) polymersome (or polymer-based vesicle), (E) dendrimersome (or dendrimer-based vesicle). For clarity, only single-bilayer vesicles were represented in this figure.

**Fig. 2:**
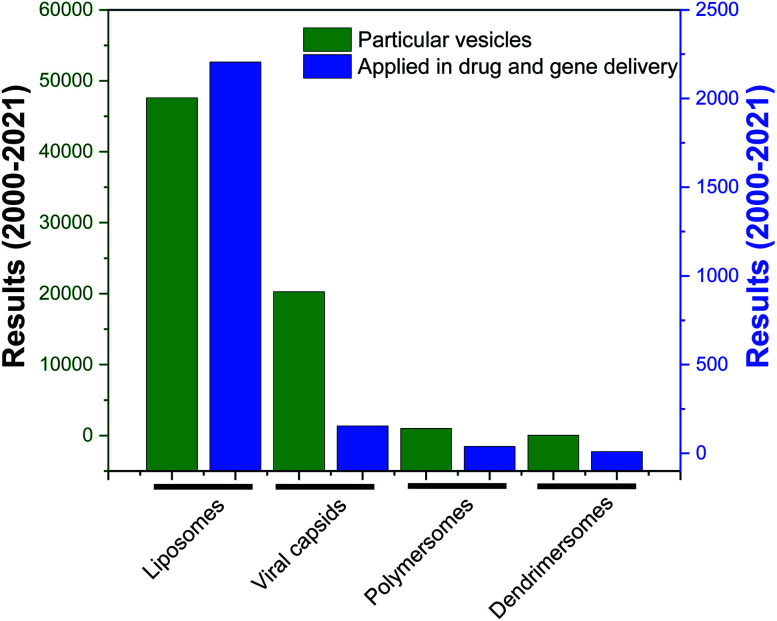
Research articles cited in PubMed (search date: 10^th^ March 2021) published from 2000 to 2021, corresponding to the keywords “liposomes”, “viral capsids”, “polymersomes”, “dendrimersomes” (for the category “particular vesicles”) and “liposomes in drug and gene delivery”, “viral capsids in drug and gene delivery”, “polymersomes in drug and gene delivery”, “dendrimersomes in drug and gene delivery” (for the category “application in drug and gene delivery”).

Over the years, the evaluation of natural and synthetic vesicles for biomedical use appeared to follow the following timeline: viral capsids (natural carriers, but immunogenic), chronologically followed by liposomes (no immunogenicity, but low stability), stealth liposomes (improved stability, but additional complexities due to the presence of several additives such as cholesterol and poly(ethylene glycol) (PEG)), polymersomes (high stability and longer circulation time, but high polydispersity and poor control over size), and finally dendrimersomes (high stability with low polydispersity, excellent physical properties in the bulk state, high degree of branching and a multiple number of end groups at the periphery) (Fig. 1 and 2).^27–31^

The formation of dendrimersomes has mainly been reported from Janus type of dendrimers for various applications over the years,^30,32–34^ which is synthetically challenging and involves more time, organic solvents and synthetic skills. On the other hand, a new generation of dendrimersomes made from cationic polyamine dendrimers is less synthetically challenging, less time consuming, requires less organic solvents and less synthetic skills. As a result, the evaluation of these vesicles was the focus of much research, including the recent development of such dendrimersomes towards anti-cancer therapy in our laboratory.

Much work has been devoted so far to the studies of polyamidoamine (PAMAM)-based amphiphilic dendrimers bearing long, soft chains at their periphery.^5,9–13,35^ By contrast, it is only recently that the aggregation behavior of another polyamine hydrophilic dendrimer, poly(propylene imine) (PPI), was investigated.^36,37^

**Fig. 3:**
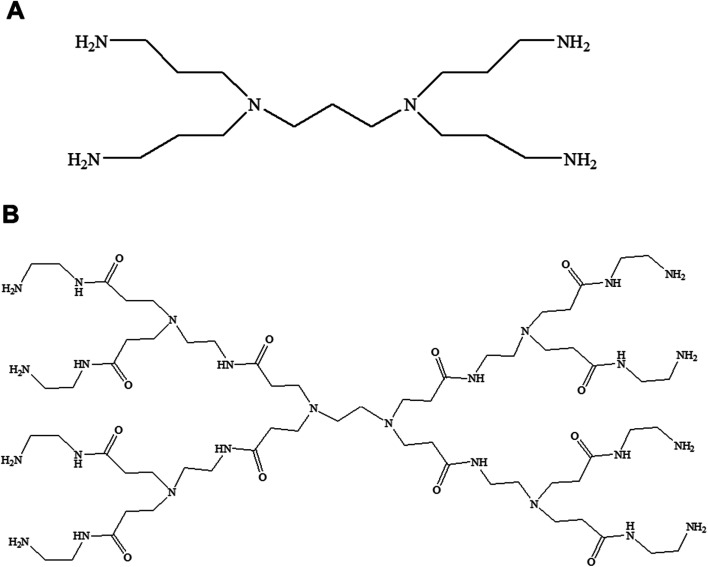
Chemical structure of generation 1-diaminobutyric polypropylenimine (PPI) (A) and generation 1-polyamidoamine (PAMAM) dendrimers (B).

**Fig. 4:**
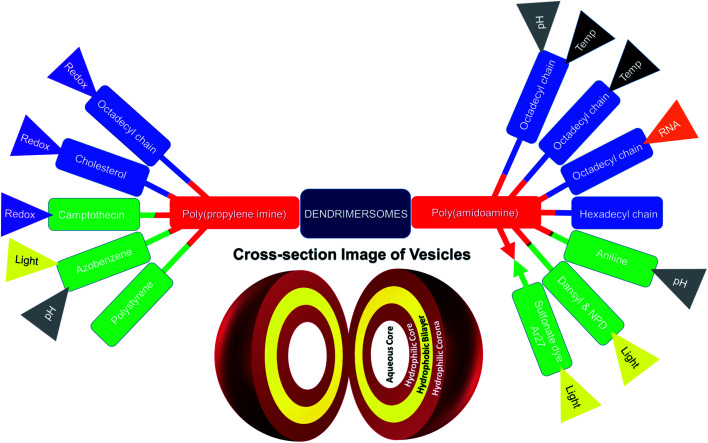
Surface-modified amphiphilic dendrimers forming stimuli-responsive and non-stimuli-responsive dendrimer-based vesicles, or dendrimersomes, presented in this review (color code for surface-modifying groups: lipids or aliphatic chains (blue), aromatic groups (green); “double colored bar”: conjugated amphiphiles or hydrophobe–dendrimer conjugate, “→←”: complexed amphiphiles or hydrophobe–dendrimer complex).

This mini-review provides an overview of the development of the self-assembled amphiphilic polyamine dendrimer-based vesicles (dendrimersomes) towards potential biomedical applications, including cancer therapy. As there is already a vast amount of literature available on the formation of self-assembled nanostructures derived from dendritic amphiphiles made from Janus type of dendrimers,^30,32–34,38–41^ our discussion will be restricted to the formation of vesicular self-aggregated structures from surface-modified dendritic polyamine amphiphilic molecules, which corresponds to a more recent development, focusing on PAMAM and PPI dendrimers, that are the most promising polyamine cationic dendrimers currently in use (Fig. 3 and 4).

## 2. PPI-based dendrimersomes: design, stimuli-sensitivity and biomedical applications

Recent developments of spontaneously formed dendrimersomes from amphiphilic PPI dendrimer after hydrophobic surface modification were reported in this part, which was subdivided based on the type of hydrophobic modification on the surface of PPI polyamine dendrimers, using various lipids and aromatic groups. Additional stimuli-responsive behavior, redox-sensitivity in particular, and gene transfection capabilities of these dendrimersomes towards their successful biomedical implication were further discussed in each of these particular sections. Generation 3 (G3)-PPI dendrimer was previously reported to be safer and more efficacious than its higher generation counterparts for gene transfection in *in vitro* and *in vivo* conditions.^42–45^ A polyethylene glycol (PEG)-based heterobifunctional cross-linker (orthopyridyl disulfide polyethylene glycol succinimidyl carboxymethyl ester OPSS-PEG-SCM) was used to conjugate the hydrophobic groups to G3-PPI dendrimer, as PEG is a biocompatible material well known to increase the water solubility of delivery systems and a stealth material to reduce non-specific interactions with circulatory proteins, thereby leading to an enhanced circulatory lifetime of drug and gene delivery systems.^26,46–49^ Our recent work also demonstrated that the conjugation of low molecular weight PEG (2 kDa) to generation 3- and generation 4-PPI dendrimers significantly decreased their cytotoxicity as compared to unmodified dendrimers, leading to increased gene expression on B16F10-Luc, A431, DU145 and PC3-Luc cells lines.^50^

### 2.1. Lipid-modified PPI dendrimers

#### 2.1.1. Octadecyl chain-modified PPI dendrimer

**Fig. 5:**
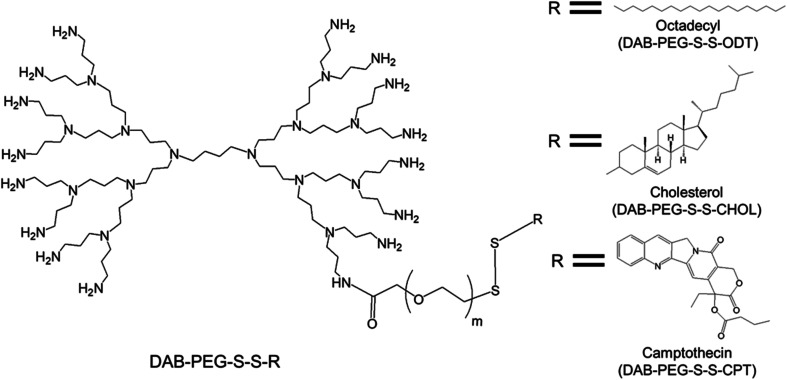
Chemical structure of an amphiphilic, surface-modified, disulphide-linked octadecyl chain-bearing/cholesterol-bearing/camptothecin-bearing PEGylated generation 3-diaminobutyric polypropylenimine (PPI or DAB) dendrimer (DAB-PEG-S-S-ODT/DAB-PEG-S-S-CHOL/DAB-PEG-S-S-CPT respectively), where *m* = 45.

We reported the spontaneous formation of dendrimersomes by octadecyl (C18) lipid chain-modified PEGylated G3-PPI dendrimer for drug and gene delivery. Being essential structural and functional hydrophobic constituents of animal cell membrane,^51–53^ long fatty acid chains have always been a preferred lipid choice for the conjugation to hydrophilic substances towards the formation of self-assembled nanostructures, as hydrophobic interactions are one of the main driving forces for any amphiphilic low molecular weight surfactants and high molecular weight polymers to self-aggregate.^54–56^ Additionally, the presence of lipid chains conjugated to the dendrimer backbone proved to enhance the overall transfection capability of cationic polymers, including polyamine dendrimers, due to the strong fusogenic activity of lipids and the formation of more compact, smaller-sized dendriplexes.^57–60^ Furthermore, transfection activity was reported to be enhanced for the octadecyl lipid-modified generation 3-PAMAM dendrimer.^61^ In line with that, we successfully synthesized disulphide-linked octadecyl (C18 alkyl) or stearyl chain-bearing PEGylated (MW 2 kDa) generation 3-PPI dendrimer, or DAB-PEG-SS-ODT (Fig. 5) through one-pot *in situ* two-step reactions, where the lipid : dendrimer ratio was nearly 1 : 1.^62^

**Fig. 6:**
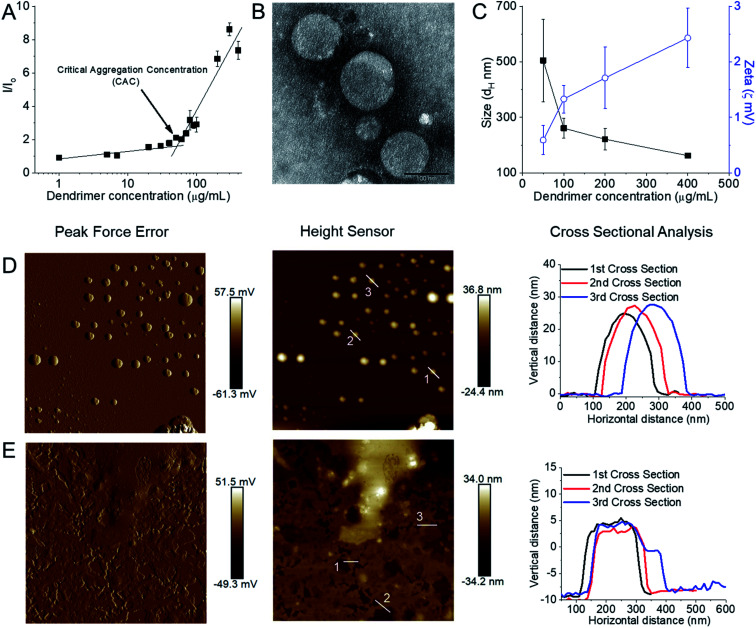
(A) Measurement of CAC from the plot of relative fluorescence intensity (*I*/*I*_0_) of a hydrophobic fluorescent dye, *N*-phenyl-1-naphthylamine in presence of various lipid–dendrimer concentration in PBS buffer (pH 7.4). (B) Stained TEM images of lipid–dendrimer based dendrimersomes (400 μg mL^−1^ in PBS, pH 7.4) (bar size: 100 nm). (C) Size (■) and zeta potential (○) of dendrimersomes at various lipid–dendrimer concentrations in PBS buffer (pH 7.4) at 37 °C. AFM peak force error and height sensor images of 5 × 5 μm surfaces of (D) 200 μg mL^−1^ and (E) 500 μg mL^−1^ dendrimersomes adsorbed on a mica (silicon) surface (reproduced from ref. 62, with permission from the journal as authors of this article).

We were unable however to synthesize octadecyl-bearing PEGylated generation 3-PPI dendrimer at other ratios of lipid : dendrimer such as 2 : 1 or 1 : 2 due to freeze thawing issues, which is a common problem for various cationic lipids and emulsion-based foods.^63,64^ Stable, cationic, nanosized vesicles were formed spontaneously from this dendrimer as a result of molecular self-assembly above its critical aggregation concentration value (∼50 μg mL^−1^) (Fig. 6A and B). The hydrodynamic diameter (*d*_H_) of these lipid–dendrimer-based dendrimersomes showed a gradual decrease in average size from low to high concentrations, due to the formation of more compact and stable nanostructures as a result of increased hydrophobic interactions among lipid parts with increasing concentration. Thus, the sizes of DAB-PEG-SS-ODT-based vesicles at various concentrations (260.8 nm ± 36.5 nm at 100 μg mL^−1^, 221.4 nm ± 39.4 nm at 200 μg mL^−1^ and 162.1 nm ± 8.1 nm at 400 μg mL^−1^) were below the cut-off size for extravasation to most tumor cells (400 nm) (Fig. 6C).^65^ DAB-PEG-SS-ODT-based vesicles were found to bear a positive zeta potential at all the tested concentrations, which increased with increasing concentrations, reaching a maximum value of 2.43 mV ± 0.54 mV at a concentration of 400 μg mL^−1^ (Fig. 6C). The positive charge on the surface of the vesicles helped to enhance the non-specific uptake of these nanostructured cationic vesicles by the cells, because of favorable electrostatic interactions between the negatively charged cell membrane and positively charged nanoparticles.^66^ The C18 lipid-bearing dendrimersome showed a crystallization exotherm at 0.2–0.3 °C and an adsorption tendency on mica surface (Fig. 6D and E). These features were more pronounced with increasing concentration of lipid–dendrimer, indicating a linear relationship of such properties with the content of conjugated lipid (Fig. 6D and E). The steady-state fluorescence intensity of emission maximum of coumarin-153 (C153) increased with increasing concentrations of dendrimersomes, suggesting an increasing hydrophobicity of the vesicular bilayer membrane with increasing lipid–dendrimer concentration resulting from a more favorable packing of amphiphiles. Overall a small blue shift (∼4 nm) of the emission maximum of C153 was observed in presence of the dendrimersomes than that in absence of dendrimersomes, indicating a comparatively polar microenvironment of this vesicular bilayer. Furthermore, the calculated micropolarity (π*-value = 0.83) of the hydrophobic microenvironment of bilayer at 1000 μg mL^−1^ lipid–dendrimer solution was found to be similar to the polarity of the polar aprotic solvent propylene carbonate (0.83).^67^ The comparatively polar microenvironment of the bilayer membrane made it less favorable for encapsulation of a substantial amount of hydrophobic drugs. Because of the presence of a disulphide linkage (–S–S–) in the backbone, this lipid–dendrimer successfully showed a redox-responsive disintegration of dendrimersomes in presence of a glutathione (GSH) concentration similar to that of the cytosolic reducing environment (10 mM GSH) in comparison to that of extracellular reducing environment (10 μM GSH) or non-reductive environment. In addition, this lipid–dendrimer was found to condense more than 70–75% of the DNA at dendrimer : DNA weight ratios of 5 : 1 and higher. Even after the dendriplex formation, the lipid–dendrimer had the capability to retain its vesicular forming properties. Dendriplexes showed less adsorption tendency on mica surface, leading to less deformation in comparison to that observed with the naked dendrimer. The cationic, stable, nanometer-sized dendrimersomes-based dendriplexes helped to enhance the cellular uptake of DNA both at dendrimer : DNA weight ratios of 10 : 1 and 20 : 1, respectively by up to 15.3-fold and 16.3-fold compared with naked DNA on PC3 prostate cancer cell line. Similarly, in DU145 prostate cancer cell line, the cellular uptake of DNA increased following treatment of the cells with this lipid-dendriplex by up to 28.6-fold and 27.6-fold compared with naked DNA. The dendriplexes showed an increased gene transfection on PC3 cell line at both dendrimer : DNA weight ratios of 10 : 1 and 20 : 1 compared with naked DNA, whereas in DU145 cells transfection was only enhanced at dendrimer : DNA weight ratio of 20 : 1. Octadecyl chain-bearing PEGylated PPI dendrimer-based (DAB-PEG-SS-ODT) dendrimersomes therefore were shown to be promising as redox-sensitive drug and gene delivery systems for potential applications in combination cancer therapy.

#### 2.1.2. Cholesterol-modified PPI dendrimer

**Fig. 7:**
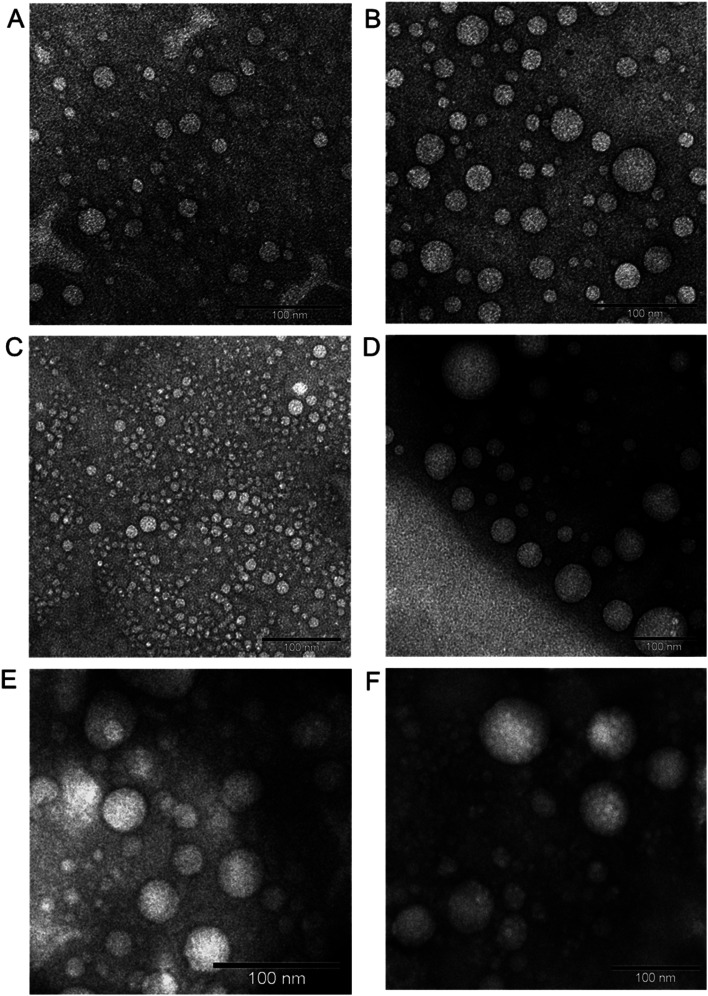
Unstained TEM images of low-cholesterol (A) and high-cholesterol (B) dendrimersomes (400 μg mL^−1^ in PBS (pH 7.4)). Unstained TEM images of dendrimersomes–DNA complexes: low-cholesterol (C and D) and high-cholesterol (E and F) dendriplexes at dendrimer : DNA weight ratios of 5 : 1 (C and E) and 10 : 1 (D and F) (reproduced from ref. 68 with permission from the journal as authors of this article).

Since hydrophobic interactions are one of the main driving forces for any aggregated structure, we have chosen to conjugate PEGylated PPI dendrimer with a more lipophilic cholesterol moiety (instead of comparatively less hydrophobic lipidic alkyl chains) to facilitate comparatively more robust and efficient self-assembly and to form more compact, smaller-sized dendriplexes.^68^ Due to its higher hydrophobicity than that of the hydrocarbon chains of fatty acids, cholesterol has been extensively utilized for the synthesis of amphiphilic low molecular weight surfactant and high molecular weight polymer-based self-assembled nanostructures.^69–71^ Literature data suggested that cholesterol not only helps to enhance the stability of the hydrophobic rigid microenvironment of nanostructures and facilitate any drug delivery systems to cross the cellular membrane, but also increase the transfection efficacy of gene vectors due to its strong fusogenic activity.^6^ In this study, we have reported the development of redox-sensitive dendrimersomes as drug and gene delivery systems comprising of disulfide-linked cholesterol-bearing PEGylated (MW 2 kDa) generation 3-PPI (or DAB-PEG-S-S-CHOL) dendrimers (Fig. 5) *via* an *in situ* two-step reaction. In this work, we have synthesized two cholesterol–dendrimers where the ratio of cholesterol (and PEG) and PPI were 2 : 1 for high cholesterol–dendrimer (12.5% conjugated cholesterol per dendrimer) and 1 : 1 for low cholesterol–dendrimer (9.5% conjugated cholesterol per dendrimer). Both of them were able to spontaneously self-assemble into stable, cationic, nanosized vesicles (Fig. 7A and B) above their critical aggregation concentrations (CAC: ∼21 μg mL^−1^ for low cholesterol dendrimer and ∼10 μg mL^−1^ for high cholesterol dendrimer). A lower CAC value for high cholesterol–dendrimer vesicles in comparison to the low cholesterol–dendrimer vesicle indicated that the overall more hydrophobic content actually facilitates the dendrimersome formation at lower lipid–dendrimer concentration. These cholesterol–dendrimers showed comparatively lower CAC values (and therefore self-assembly formation at lower lipid–dendrimer concentration) than that of the C18 alkyl chain modified dendrimers (mentioned in the previous section), due to the higher hydrophobicity of cholesterol than the alkyl chain. Both low- and high-cholesterol dendrimer-based dendrimersomes displayed an average size less than 230 nm with low polydispersity index (PDI) values at all the tested concentrations, with a gradual decreasing trend from low to high concentrations. They showed the smallest sizes of 91.3 nm ± 11.3 nm and 133.4 nm ± 48.8 nm for low- and high-cholesterol dendrimer-based dendrimersomes at 400 μg mL^−1^ (among tested concentrations) respectively. The formation of more compact and stable dendrimersomes at higher dendrimer concentrations due to increased hydrophobic interactions among cholesterol was the reason behind such a decrease in size. Both the dendrimersomes showed positive zeta potential between 4 mV and 7 mV with an increasing trend with increasing concentrations. The maximum zeta potential values of 5.7 mV ± 0.5 mV and 6.5 mV ± 0.3 mV was observed at 400 μg mL^−1^ (among tested concentrations) for low- and high-cholesterol dendrimer-based dendrimersomes, respectively. The size and zeta potential of low- and high-cholesterol dendrimersomes were not statistically different at all the tested concentrations with the change of the amount of cholesterol within the dendrimers. TEM images of the dried samples on carbon coated copper grid revealed the formation of spherical unilamellar vesicles of sizes less than 50 nm. These dendrimersomes showed a successful entrapment of hydrophilic and hydrophobic dyes. Both of them showed no lower critical solution temperature (LCST) or cloud point (CT) in the temperature ranging from 20 to 90 °C, facilitating their no phase separation of vesicles at body temperature (37 °C), and therefore no premature release of any entrapped pharmaceutically active agent at body temperature. In DSC study, both the dendrimersomes showed an endothermic melting peak (*T*_m_) due to the phase change of the vesicle membrane from the “gel” state to the “liquid crystalline” or “fluid” state at nearly 0 °C (−0.6 °C and −0.8 °C for low- and high-cholesterol dendrimer-based vesicles, respectively) during the first heating phase and second heating cycle. Such a low *T*_m_ values for these dendrimersomes might be due to the presence of unsaturation in cholesterol and/or in the disulfide bond. AFM images showed that both dendrimer-based vesicles adsorbed on a freshly cleaved mica surface after air-drying, leading to the diffusion of the vesicles. Due to the presence of disulfide-linked hydrophobic cholesterol in the bilayer of dendrimer-based vesicles, they showed redox-sensitive properties in presence of intracellular reducing environment (10 mM GSH). It was found that both the dendrimersomes showed a gradual increase in size over time in presence of a higher concentration (10 mM) of GSH in comparison to that in the presence of 10 μM or without GSH, which can be attributed to the cleavage of the disulfide bonds and disintegration of vesicles, forming larger, unstable and less compact aggregates in intracellular redox condition. Both dendrimersomes showed a higher amount of cumulative release of entrapped hydrophobic Nile red over time from the vesicles in in the presence of a glutathione concentration similar to that of a cytosolic reducing environment (up to 40% and ∼25% Nile red release from low- and high-cholesterol dendrimersomes respectively in the presence of 10 mM GSH) rather than in extracellular redox condition. Thus the disassembly of the vesicles and concomitant release of the model drug in the intracellular or cytosolic reducing environment (corresponding to 10 mM GSH) proved the possibility of future use of these dendrimer-based vesicles as redox-responsive drug delivery systems for cancer treatment. The high-cholesterol dendrimersomes showed higher melting enthalpy (in DSC study), increased adsorption tendency on mica surface (in AFM study), have higher entrapment ability for hydrophobic drugs and displayed an increased resistance to redox-responsive environments in comparison with their low-cholesterol counterpart due to a progressive “dilution” of the lipids (cholesterol) in the bilayer membrane from high- to low-cholesterol dendrimer-based vesicles. Both dendrimersomes were able to condense plasmid DNA quite efficiently along with the retention of their vesicular structures after complexation with DNA (Fig. 7C–F). Low-cholesterol vesicles were able to condense more than 85% of the DNA at all the tested dendrimer : DNA weight ratios, whereas high-cholesterol vesicles having fewer primary amine at the periphery, showed condensation ability over 85% at dendrimer : DNA weight ratios of 1 : 1 and higher. Due to the presence of highly hydrophobic cholesterol in the backbone, these dendriplexes showed an overall smaller hydrodynamic diameter, leading to the formation of more compact dendriplexes in comparison to not only DAB-PEG-S-S-ODT-based dendrimersomes (described in the previous sections), but also the unmodified DAB dendriplex. Both dendrimers showed positive surface charge ranging between 21.4 ± 0.9 and 41.1 ± 0.6 mV for most dendrimer : DNA weight ratios from 1 : 1 and above. Further, both dendriplexes retained the vesicular morphology after complexation with DNA, as evidenced from the TEM images of dendriplexes at dendrimer : DNA weight ratios of 5 : 1 and 10 : 1. Thus, positively charged and smaller-sized compact dendrimersome-based dendriplexes enhanced the cellular uptake of DNA through internalization mechanisms by up to 8–12-fold and 10–15-fold when compared with unmodified G3-PPI dendriplex and naked DNA respectively. Overall, these dendriplexes helped to increase the gene transfection in the PC-3 prostate cancer cell line at various dendrimer : DNA weight ratios, by about 5-fold compared with the gene expression observed following treatment of the cells with naked DNA. These cholesterol-bearing PEGylated dendrimersomes were more promising as redox-sensitive drugs and gene delivery systems than the C18 alkyl-bearing PEGylated dendrimersomes.

### 2.2. Aromatic group-modified PPI dendrimers

#### 2.2.1. Camptothecin (quinoline alkaloid)-modified PPI dendrimer

**Fig. 8:**
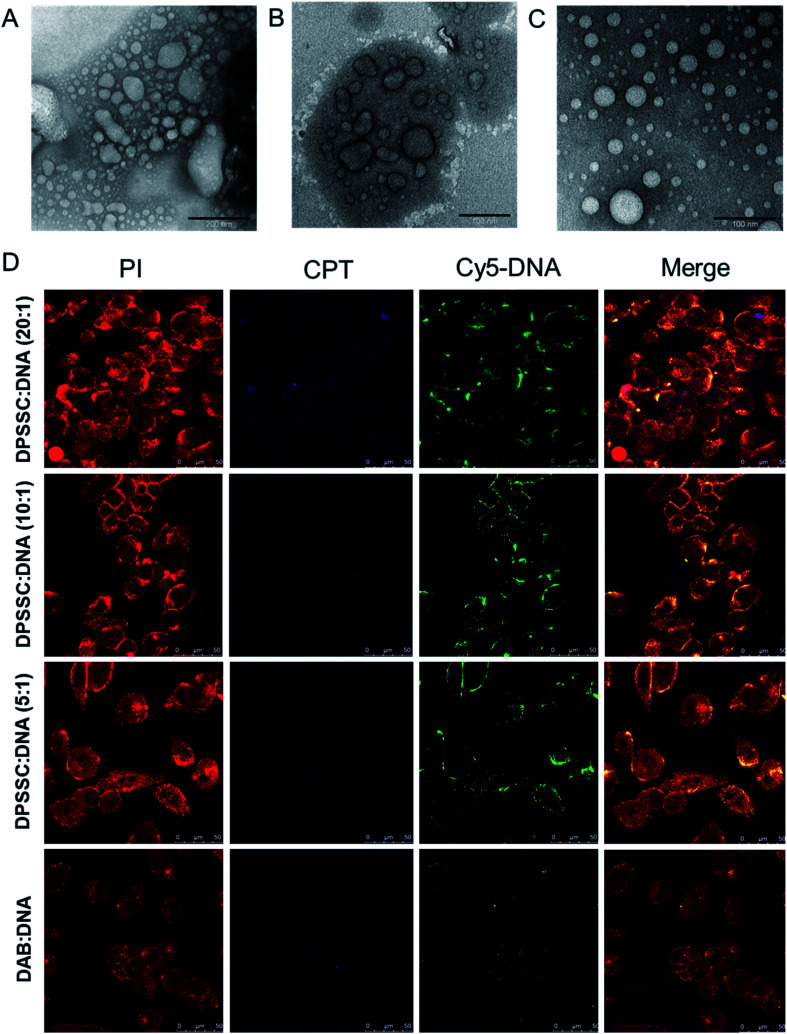
TEM images of dendrimersomes formed by CPT–dendrimer solutions (pH 7.4) at various concentrations: 0.2 mg mL^−1^ (A), 1.0 mg mL^−1^ (B) and 2.0 mg mL^−1^ (C) (scale bar: 100 nm). (D) Confocal microscopy images of the cellular uptake of CPT–dendrimer conjugate complexed with Cy5-labelled DNA (2.5 μg per well) at various weight ratios (control : DAB dendrimer : DNA) (magnification: ×40) (reproduced from ref. 72 with permission from the journal as authors of this article).

In spite of their advantages regarding the combination delivery of drugs and genes, cholesterol-based dendrimersomes (mentioned in Section 2.1.2.) may face the undesired release of the entrapped drugs before reaching their target site of action. To overcome this potential issue associated with the physical entrapment of drugs in the delivery systems, we have designed and synthesized a pro-drug dendrimer by conjugating the anti-cancer drug camptothecin (CPT) with PEGylated generation 3-diaminobutyric polypropylenimine dendrimer (or DAB-PEG-S-S-CPT) through a redox-sensitive disulphide linkage (Fig. 5) in a similar fashion as DAB-PEG-S-S-ODT and DAB-PEG-S-S-CHOL.^72^ The ratio of CPT (and PEG) and PPI was found to be nearly equal to 1 : 1 for the synthesized CPT–dendrimer, DAB-PEG-S-S-CPT. This PEGylated pro-drug dendrimer was found to spontaneously self-assemble into cationic vesicles at pH 7.4. It formed the dendrimersomes above a comparatively higher critical aggregation concentration (∼200 μg mL^−1^) than that of lipid–dendrimer-based dendrimersomes due to the lower hydrophobicity of CPT than that of lipid. The hydrodynamic size of dendrimersomes measured at various tested concentrations (0.5, 1 and 2 mg mL^−1^) showed a sharp decrease with time and increasing concentrations. The hydrodynamic sizes became steady after 12 h at 37 °C and remained stable at around 250–300 nm (for 0.5 mg mL^−1^) and 150–200 nm (for 1 and 2 mg mL^−1^), leading to the formation of more stable and compact nanostructures at higher concentrations due to increased hydrophobic interactions among CPT molecules. The size of dendrimersomes in presence or absence of complete medium containing 10% FBS (size ∼ 100 nm) was below the cut-off size for extravasation. The formation of stable cationic dendrimersomes at various tested concentrations (0.5–2 mg mL^−1^) was evidenced from the positive zeta potential (∼3–5 mV) for up to 7 days. TEM images revealed the unilamellar, spherical-shaped vesicles (dendrimersomes) with a size lower than 100 nm (Fig. 8A–C). The micropolarity (π*-value = 0.278) value obtained using coumarin-153 (C153) probe study suggested that the polarity of hydrophobic bilayer microenvironment was similar to the polarity of hexafluorobenzene (0.27), confirming that the vesicular bilayer was composed of the aromatic benzene rings of CPT. The appearance of the shoulder band in the fluorescence spectrum of C153 indicated a distribution of probe molecules at multiple locations in vesicular assemblies. Circular dichroism (CD) spectra of dendrimersomes (2 mg mL^−1^) in phosphate buffer (pH 7.4) suggested a chiral packing of the CPT moieties in the vesicular bilayer with negative chirality and left-handed helical arrangement. The CPT release from dendrimersomes (1 mg mL^−1^ in phosphate buffer, pH 7.4) was found to be around 10 to 15% in non-redox and extracellular redox condition in normal cells (in presence of 10 μM GSH). It sharply increased with time to about 30–35% within 7 days in presence of 10 mM GSH (equivalent to the intracellular concentration in normal cells) and 70% in presence of 50 mM glutathione (equivalent to the intracellular environment of tumor tissue) due to the disintegration of dendrimersomes. Additionally, the pro-drug dendrimer could instantly condense more than 85% of the DNA at dendrimer : DNA weight ratios of 5 : 1 and higher, forming stable vesicular aggregates over time. Such stable and cationic dendrimersome–DNA complexes at dendrimer : DNA weight ratios of 5 : 1 and higher led to an enhanced cellular uptake of DNA (by up to 1.6-fold) and increased gene transfection (by up to 2.4-fold) in prostate cancer cells in comparison with the unmodified G3-PPI dendrimer (Fig. 8D). These redox-responsive camptothecin-bearing cationic dendrimersomes are therefore promising carrier-based delivery systems towards combination cancer therapy involving chemotherapy along with gene therapy. Further, such a strategy could also be used for synergistic combination of drugs and nucleic acids of interest against any particular cancer. Moreover, due to the capability of these drug-conjugated dendrimersomes to entrap various hydrophobic and hydrophilic guests in their hydrophilic aqueous core or hydrophobic bilayer, such dendrimersomes could be used for theranostic applications by delivering imaging agents in addition to therapeutic drugs and nucleic acids to the disease site.^73–75^

#### 2.2.2. Azobenzene- and palmitoyl-modified PPI dendrimers

**Fig. 9:**
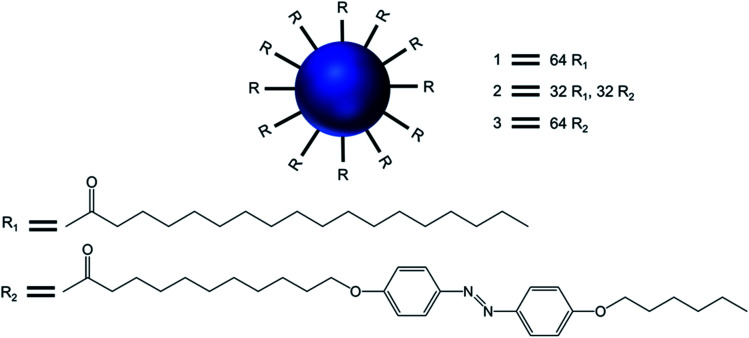
Chemical structure of the surface-modified dendritic amphiphiles made of generation 5-poly(propylenimine) dendrimers 1, 2, and 3, with the dendrimers decorated with side chains containing 64 palmitoyl, 32 palmitoyl, 32 azobenzene and 64 azobenzene groups, respectively.

Tsuda and colleagues reported in 2000 the formation of giant micrometer-sized spherical vesicles with a multilamellar “onion-like” structure in aqueous solution below pH 8 from generation 5-PPI dendrimer functionalized with aliphatic chains.^37^ Their study described the synthesis and characterization of three surface-modified generation 5-PPI dendrimers 1, 2, and 3, decorated with side chains containing 64 palmitoyl, 32 palmitoyl and 32 azobenzene, and 64 azobenzene groups respectively (Fig. 9).

Transmission electron microcopy and confocal fluorescence microscopy experiments demonstrated the formation of dendrimer-based giant vesicles in aqueous dispersion. Additionally, they demonstrated that the azobenzene-functionalized vesicles (2 and 3) were able to fluoresce, with a maximum at *λ*_max_ 600 nm, due to the dense and ordered arrangement of the azobenzene chromophore in the hydrophobic bilayer of the vesicular aggregates. Hydrogen bonds between protonated cores of the dendrimers (at pH < 8) and the π–π stacking of the azobenzene moieties inside the bilayer (as evidenced by the emission of the vesicles) were the main factors involved in the stabilization of such micrometer-sized vesicles. The vesicular structure and size distribution were found to be dependent on the end groups at the periphery of the dendrimer and the pH of the solution. Giant vesicles 2 and 3 have their morphology altered along with the refractive index of the solution, upon exposure to laser irradiation with 1064 and/or 420 nm light, as (i) infrared light induced a structural rearrangement, and (ii) 420 nm light induced a *cis*–*trans* isomerization of azobenzene units, leading to structural changes accompanied by an increase in the emission intensity. Further, vesicles in an aqueous solution at pH 1 showed an increase in fluorescence intensity with a blue shift of the emission maximum to 540 nm (due to higher degree protonation of the vesicles only after illumination), whereas this blue shift was not observed for vesicle solutions in Milli-Q® water at pH 5.5. The enhanced fluorescence intensity due to the reorganization of the chromophores within the giant vesicles was attributed to the fact that the kinetically formed giant vesicular systems in which the packing of the liquid-crystalline azobenzene units were not optimal, changed to a thermodynamically more relaxed state after light-induced isomerization.

#### 2.2.3. Polystyrene-modified PPI dendrimer

**Fig. 10:**
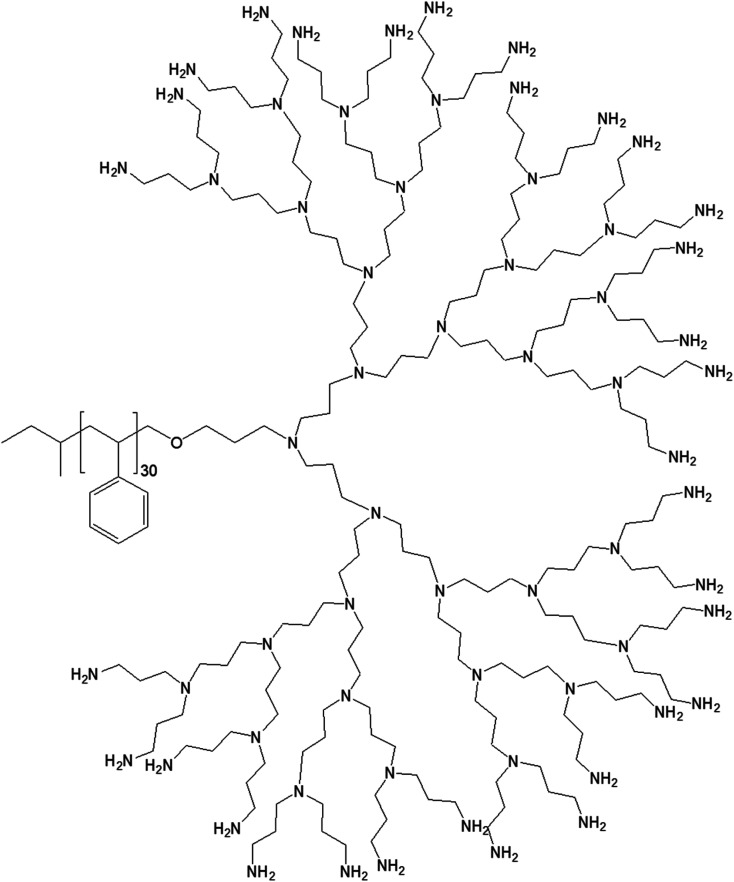
Chemical structure of polystyrene conjugated to the highest generation poly(propylene imine) dendrimer PS-dendr-(NH_2_)_32_.

In 1995, Meijer group reported the formation of novel amphiphilic macromolecules resulting from the conjugation of well-defined polystyrene (PS) moiety to PPI dendrimers of 5 generations (Fig. 10).^76^

PS-dendr-(NH_2_)_32_, PS-dendr-(NH_2_)_16_ and PS-dendr-(NH_2_)_8_ respectively formed spherical micelles, micellar rods and vesicular structures in aqueous phase, as demonstrated by dynamic light scattering, conductivity measurements and transmission electron microscopic studies. The lower generations of this class of amphiphilic macromolecules showed inverted micellar behavior in toluene, which was predominantly determined by the apolar PS chain. Conductivity measurements were used to assess the amphiphilic properties of PS-dendr-(NH_2_)_*x*_ in toluene–water mixtures as a function of the toluene/water ratio, which actually helped to identify the point of phase inversion from water to toluene as the continuous phase. All lower generation amphiphiles displayed a strong tendency to stabilize toluene as the continuous phase. The highest inversion point was observed for PS-dendr-(NH_2_)_16_, which was well beyond 50% toluene in volume. Further, the inversion point of PS-dendr-(NH_2_)_32_ could not be determined, due to the dendrimer insolubility in toluene. Based on the differences in phase inversion from water to toluene, the authors suggested various types of aggregation pattern by these amphiphiles due to their different amphiphilic behavior. The DLS measurements was not very confirmatory, especially for higher generation of dendrimer, due to the clustering between the aggregates. Finally, TEM study using three different techniques (negative staining with uranyl acetate, Pt shadowing, and freeze fracture) showed a clear change in aggregation pattern from PS-dendr-(NH_2_)_8_ to PS-dendr-(NH_2_)_32_, from vesicles [for PS-dendr-(NH_2_)_8_] to micellar rods [for PS-dendr-(NH_2_)_16_, 12 nm in diameter], to spherical micelles [for PS-dendr-(NH_2_)_32_, 10 to 20 nm in diameter], which is in qualitative agreement with the theory of Israelachvili and colleagues on the relation between the type of aggregation and the amphiphile molecular shape.^77^ Thus, the size of the amphiphilic dendrimer head group played an important role to form such various aggregates, from spherical to inverted micelles. The vesicular structures formed by PS-dendr-(NH_2_)_8_ showed a resemblance to the structure proposed by Kunitake and colleagues.^78^ Their diameters (10 to 20 nm) were in the same order of magnitude as the estimated bilayers of the PS-dendrimer block copolymers. These amphiphiles were reported to be closer in shape and in size as compared with traditional surfactants and traditional block copolymers respectively.

## 3. PAMAM-based dendrimersomes: design, stimuli-sensitivity and biomedical applications

In this part, we have reported recent developments of spontaneously and non-spontaneously (stimuli-sensitive) formed dendrimersomes from amphiphilic PAMAM dendrimer following hydrophobic surface modification. This section was similarly subdivided based on the type of hydrophobic modification on the surface of PAMAM polyamine dendrimers using various lipids and aromatic groups.

### 3.1. Lipid-modified PAMAM dendrimers

#### 3.1.1. Octadecyl alkyl chain-modified PAMAM dendrimer

##### 3.1.1.1. pH-Responsive dendrimersomes

**Fig. 11:**
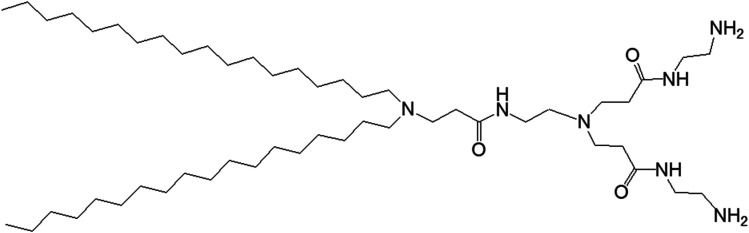
Chemical structure of amine-terminated, generation 1-polyamidoamine dendron bearing two octadecyl hydrophobic chains.

In 2015, Doura and colleagues reported the synthesis and characterization of pH-responsive lipid-bearing PAMAM dendron-based vesicular assemblies, formed following conjugation of two octadecyl hydrophobic chains to amine-terminated generation 1-(G1) polyamidoamine dendron (Fig. 11).^11^

The presence of two primary amines and two tertiary amines in the lipid–dendron moiety made this dendrimer particularly promising due to its dynamic molecular shape at various pHs. It was able to form vesicle assemblies at neutral and alkaline pHs, but its structure changed to a micelle-like structure below pH 6.4. DSC analysis of this lipid–dendrimer showed a sharp endothermic peak (around 43 °C) at pH 10 and 7.4, but a broader endothermic peak with a gradual shift to a lower temperature at pH below 6.0, indicating the formation of a bilayer structure with a gel-to-liquid crystalline transition around 43 °C under neutral and alkaline conditions but not in acidic condition. The pH-responsive property of the lipid–dendrimer-based vesicles was confirmed by DLS study. The hydrodynamic size of the vesicles was around 75 nm at pH 7.4, whereas their diameter decreased to around 20 nm below pH 6.5 after a structural shift from vesicles to micelles. This was confirmed further by TEM imaging, which also showed the formation of vesicles with a diameter around 100 nm at pH 7.4, and the formation of micelles with diameter less than 20 nm at pH 6.5 and pH 4.0. Furthermore, the lipid–dendron amphiphile also produced sharp pH-responsive vesicles with high colloidal stability in presence of PEG-lipid, which eventually transformed to micelle-like structure at around pH 6.5. The PEG-incorporated lipid–dendron vesicles were able to encapsulate FITC-labeled ovalbumin (a water-soluble protein found in egg white, with a molecular weight of 45 kDa) in their internal aqueous space, and also acid-triggered its almost complete release at pH 6.0. An efficient ovalbumin delivery from PEG-incorporated dendron–lipid vesicles was also observed into the cytosol of DC2.4 mouse dendritic cells after internalization through endocytosis. These pH-responsive dendrimersomes showed a promising efficacy for cytoplasmic delivery of bioactive molecules, such as proteins, indicating their future potential towards pH-responsive cancer therapy.

##### 3.1.1.2. Temperature-responsive dendrimersomes

**Fig. 12:**
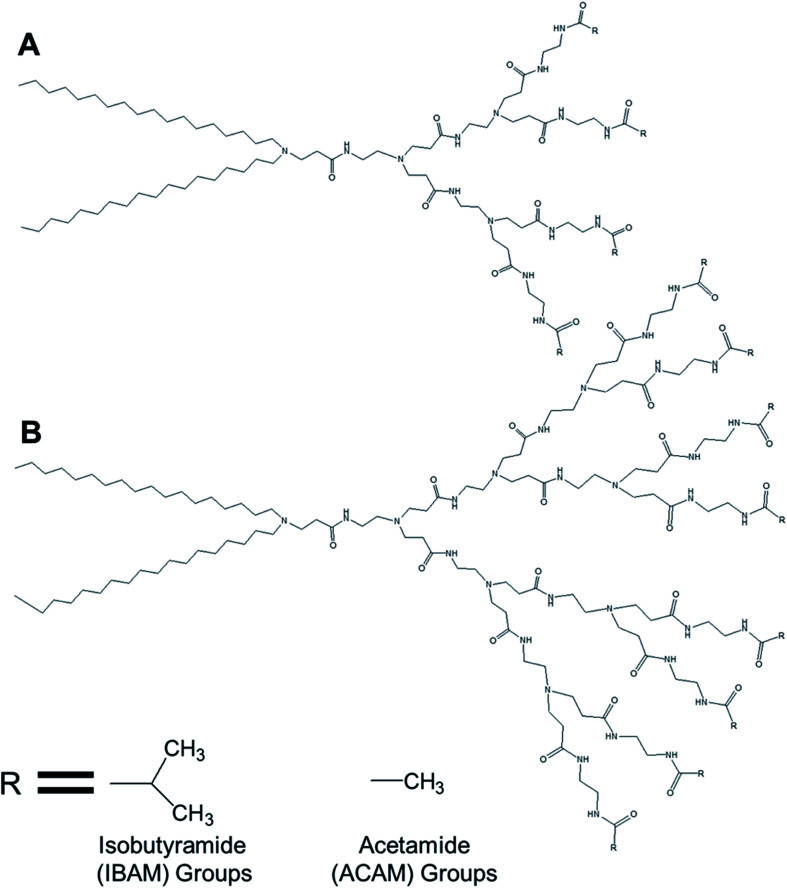
Chemical structure of (A) generation 2- and (B) generation 3-PAMAM dendrimer conjugated to two octadecyl chains (R = isobutyramide (IBAM) or acetamide (ACAM) terminal groups).

Kono and colleagues reported, in 2011, the formation of temperature-sensitive vesicles by a series of alkyl amide-PAMAM dendrons with varying chain terminal moiety that undergoes a temperature-dependent transition through a change in hydration of the vesicle surface.^35^ They synthesized PAMAM-based lipid–dendrimers by conjugating amine groups of generation 2- (G2) and generation 3- (G3) PAMAM dendrons with two octadecyl chains, where the dendrimer and the lipid chains were used as head group and hydrophobic tails, respectively (Fig. 12). They also made these lipid–dendrimers temperature-sensitive by modifying the thermosensitive isobutyramide (IBAM) groups at every chain terminal of the dendron moiety to produce two IBAM-based thermos-sensitive dendrimers, IBAM-G2-DL and IBAM-G3-DL. To correlate their assumption on the impact of the terminal groups with the change of temperature, they also prepared lipid–PAMAM dendrimer with acetamide (ACAM) groups at every chain terminal of the dendron moiety, ACAM-G2-DL and ACAM-G3-DL.

The authors successfully demonstrated the temperature sensitization of lipid-bearing dendrimer-based vesicles. Temperature-sensitive IBAM-terminated lipid–dendrimers showed an increase in the cloud point with decreasing pHs of the dispersions, due to the protonation of the tertiary amines of the PAMAM dendrimer leading to the enhanced hydration of the conjugated lipid moiety at lower pH values. Transmission electron microscopy confirmed the vesicular morphology of IBAM-G2-DL assemblies in aqueous solution, with a diameter of 50–100 nm at 15 °C. However, these vesicular assemblies were changed to fibrous structures about 8–10 nm in thickness after 10 min incubation at 50 °C (higher than the cloud point of IBAM-G2-DL). By contrast, the IBAM-G3-DL molecules not only formed spherical vesicles with diameters of 100–200 nm at 15 °C, but also were able to retain their spherical vesicular morphology after 10 min incubation above the cloud point, indicating a fusion of vesicles during the incubation. Such a morphology transition phenomenon of these lipid–dendrimer-based vesicular assemblies below and above the cloud point was also supported by DLS, AFM and SAXS profiles at various temperatures. In DSC measurements, ACAM-G2-DL and ACAM-G3-DL displayed no endotherm, whereas the IBAM-G2-DL and IBAM-G2-DL dendrimers showed clear endothermic peaks in the same temperature region. These DSC results demonstrated the importance of the dehydration of the IBAM groups on the vesicle surface for the generation of such endothermic peaks. Additionally, a concomitant increase of cloud point with increasing ACAM content proved the enhanced hydration of the surface of the assemblies due to the inclusion of hydrophilic ACAM groups. Thus, in contrast to IBAM-G2-DL, ACAM-G2-DL dispersion was found to be stable under the experimental conditions.

##### 3.1.1.3. Adaptive dendrimersomes upon interaction with siRNA

**Fig. 13:**
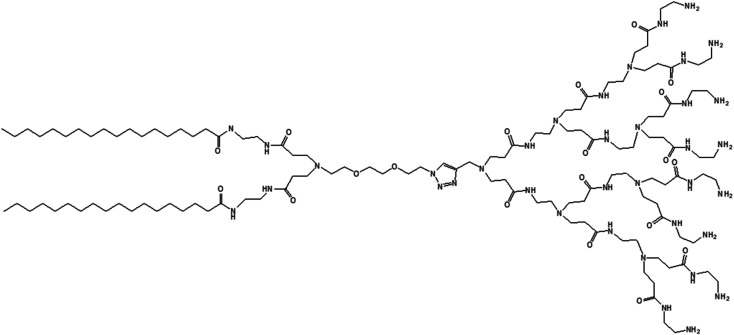
Chemical structure of lipid-modified amphiphilic PAMAM dendrimer (AD) obtained by conjugating two precursors (azide-functionalized hydrophilic dendrimer moiety and alkyne-functionalized C-18 alkyl bearing hydrophobic moiety) through click chemistry.

Liu and colleagues reported, in 2014, the formation of C-18 alkyl chain-modified amphiphilic PAMAM dendrimer-based dendrimersomes that were able to spontaneously re-assemble into comparatively smaller-sized spherical micelles upon interaction with siRNA.^79^ The amphiphilic, adaptive PAMAM dendrimer (AD) was synthesized by conjugating two precursors (azide-functionalized hydrophilic dendrimer moiety and alkyne-functionalized two C-18 alkyl-bearing hydrophobic moiety) through click chemistry (Fig. 13).

The amphiphilic AD could self-assemble in water into spherical unilamellar vesicles of about 200 nm in diameter, as demonstrated by DLS and TEM studies. Additionally, TEM images showed a bilayer structure (corona of the vesicles) of 7 nm thickness, which is approximately twice the theoretical length of AD (∼3.5 nm). Furthermore, the formation of Langmuir monolayer film at the air/water interface indicated a lipid-like behavior and vesicle-forming properties of amphiphilic AD. The authors observed an adaptive supramolecular assemblies formed by AD upon interaction with siRNA, where AD-dendrimersomes underwent structural rearrangement to form nanosized siRNA/AD complexes. Such siRNA/AD complex-based spherical substructures of 6–8 nm in diameter (twice the size of AD) indicated the formation of micellar nanostructures. This structural transition from the larger-sized vesicles into smaller-sized micelles upon complexation with siRNA helped to expose the positively charged dendrimer surface area, therefore providing better stabilization of complexes through electrostatic interactions with the negatively charged siRNA. Additional mesoscale computational studies using dissipative particle dynamics (DPD) supported that AD self-assembled into large supramolecular structures, and planar section demonstrated the formation of double-layered dendrimersomes. The presence of siRNA actually destabilized the balance between the two opposing forces, hydrophobic interactions among conjugated alkyl chains (favorable for cooperative construction of the supramolecular assemblies), and repulsive interactions/steric effects among the positively charged head groups of amphiphilic dendrimers, leading to structural changes. These stable siRNA–AD nano-assemblies effectively delivered siRNAs to various cell lines in comparison to the commercially available vector Lipofectamine RNAiMAX® (lipo), including PBMC-CD4+ human primary peripheral blood mononuclear cells, HSC-CD34+ hematopoietic stem cells and glioblastoma stem cells (GSCs) towards the development of successful non-viral vectors for delivery of RNAi therapeutics. These siRNA–AD nanocomplexes were resulted in 50% reduction of viral infection in both PBMC-CD4+ and HSC-CD34+ cells. Furthermore, the significant reduction of gene silencing ability of AD/siRNA complex in the presence of bafilomycin A1 (a proton pump inhibitor) proved that the proton sponge effect (protonation of tertiary amines in the dendrimer interior leading to endosomal disruption and subsequent cargo release in the cytoplasm) was highly important for successful AD-mediated siRNA delivery.

#### 3.1.2. Hexadecyl or C16 (fatty acid) chain-modified PAMAM dendrimer

##### 3.1.2.1. Balance of hydrophobic and hydrophilic groups

**Fig. 14:**
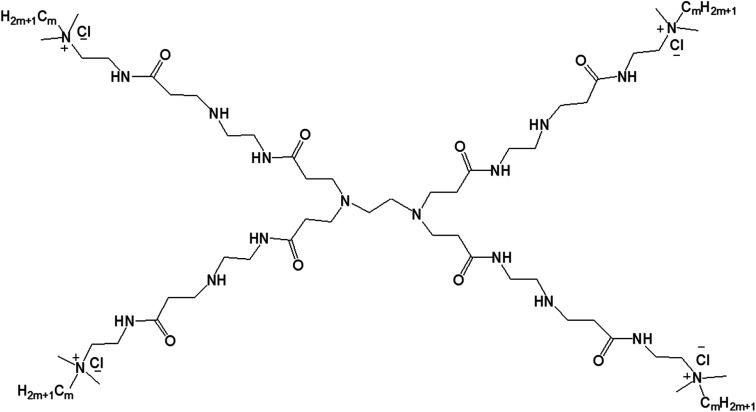
Chemical structure of amphiphilic lipid-modified PAMAM dendrimers G_*n*_QPAMC_*m*_ (where *n* = 1 (dendrimer generation) and *m* = 8, 12, 16 (alkyl chain length after the modification of the end group)).

In 2015, Zhang and colleagues reported the self-aggregation of a series of amphiphilic G_*n*_QPAMC_*m*_ (where *n* = 1 (dendrimer generation) and *m* = 8, 12, 16 (alkyl chain length after the modification of the end group)) lipid-modified PAMAM dendrimers, formed by the conjugation of the same hydrophilic group (generation-1 PAMAM) to alkyl groups of various chain length in aqueous solution (Fig. 14).^13^

The authors observed significant differences in the morphology of self-aggregates of amphiphiles with their chemical structures or with the change of hydrophobic and hydrophilic balance of amphiphiles, more specifically with the chain length of alkyl groups attached to the dendrimer surface. Cryo-TEM images confirmed the formation of spherical aggregates with a particle size of 50–100 nm for all G_1_QPAMC_*m*_, which was in agreement with DLS results. The aggregates of G_1_QPAMC_8_ were irregularly spherical, unlike the regular spherical shape observed for those made of G_1_QPAMC_12_ and G_1_QPAMC_16_. Additionally, only G_1_QPAMC_16_ (with an alkyl chain length of 16) was able to form 50–100 nm vesicles with clear boundaries in comparison to the other two amphiphiles G_1_QPAMC_8_ and G_1_QPAMC_12_. Critical micelle concentration was found to decrease with the increase in chain length, though not linearly indicating a differentiation in the morphology. With the help of fluorescence studies using two hydrophobic fluorescent probes pyrene and DPH, the authors revealed (i) a lower polarity, (ii) higher solubilization ability of microdomains of such self-aggregates and (iii) enhancement of the hydrophobic microenvironment with the increase in alkyl chain length from C8 to C12. In contrast, for G_1_QPAMC_16_ amphiphile, they observed an increase not only in the polarity of the microdomain of aggregates (in comparison to that of G_1_QPAMC_12_) but also a sharp hydrophobic probe solubilization ability, indicating a boundary structure (bilayer or multilayer) within the vesicular aggregates. The thermodynamic properties and related parameters also helped to understand the formation of aggregates with various morphologies. The enthalpies of micelle formation (or Δ*H*_mic_ values) for G_1_QPAMC_8_, G_1_QPAMC_12_, and G_1_QPAMC_12_ were −2.3, −8.5, and −5.2 kJ mol^−1^, respectively, indicating that the enthalpy and entropy jointly contributed in the self-assembly process of the amphiphilic G_1_QPAMC_*m*_. An enhancement of absolute value of the enthalpy, from −2.3 kJ mol^−1^ (G_1_QPAMC_8_) to −8.5 kJ mol^−1^ (G_1_QPAMC_12_), proved the dominating impact of enhanced hydrophobic interactions with the increase in alkyl chain length, whereas for G_1_QPAMC_16_, the decrease in the absolute value of the enthalpy indicated an opposite feature. It was found that the ratios of theoretical alkyl chain length/radius are less than one for G_1_QPAMC_8_ (10.16/19.77) and G_1_QPAMC_12_ (15.24/19.77), and higher than one for G_1_QPAMC_16_ (20.32/19.77), indicating that the head group of such amphiphiles also played an important role along with the hydrophobic interactions in the self-aggregation process and thereby enthalpy changes. Such an alkyl chain length/radius value (higher than 1) for G_1_QPAMC_16_ led to the conclusion that the intra-cohesion of the alkyl chain (C16) was involved in the process of self-assembly of the lipid-modified dendrimer, which resulted in the weakening of the hydrophobic interactions and thereby in the increase in −Δ*H*_mic_.

### 3.2. Aromatic group-modified dendrimers

#### 3.2.1. Aniline pentamer (AP) modified-dendrimer

**Fig. 15:**
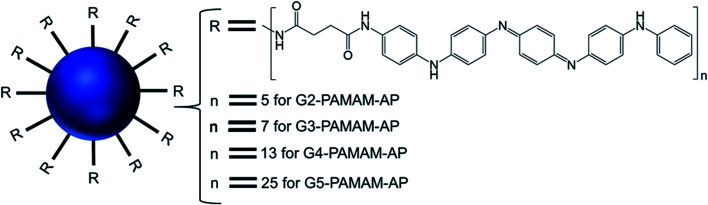
Structure of aniline pentamer (AP) conjugated to various generations (G2–5) of PAMAM dendrimer, forming PAMAM-AP amphiphiles (where R = aniline pentamer, *n* = average number of AP groups).

Hung and colleagues reported the formation of bilayer vesicular assembly from a series of PAMAM dendrimer-based amphiphiles, synthesized by conjugating various generations (G2–G5) of hydrophilic PAMAM dendrimer with a hydrophobic shell of aniline pentamer (AP) (Fig. 15).^10^

The CAC of such AP-modified dendrimers self-assembled to vesicular aggregates in water were not only very low (10^−6^–10^−7^ M), but also decreased with increasing generation of PAMAM. This trend suggested that the formation of vesicles is favored in the order G5 > G4 > G3 > G2, as the higher-generation (G4 and G5) amphiphilic PAMAM-AP dendrimers holds stronger self-organization tendency due to the π–π interactions among AP units and the H-bonding interactions between the dendrimer branches. The TEM images of PAMAM-AP G2–5 demonstrated the formation of 100–200 nm spherical vesicular aggregates, with gradually decreasing size of the bilayer vesicles as the dendrimer generation increased from G2 to G5, due to the loosely held nanostructures for the lower-generation dendrimers. These vesicles were very adhesive due to the presence of flexible hydrophilic dendritic branches in the outer layer, leading to a stronger self-adhesion *via* H-bonding interactions of the branches for the higher-generation dendrimers than for the lower-generation counterparts. Moreover, due to the strong π–π interactions of the long hydrophobic rigid AP units in the self-assembled nanoarchitectures, the average thickness of the hydrophobic interphase was reduced to 1.7–2.1 nm, which is well below the head-to-head theoretical distances (4.8 nm) of the AP double layer. Stained TEM images with uranyl acetate showed exactly reversed images in comparison to the unstained images, as the outer and inner layers of the vesicles appear in black with negative staining leading to the conclusion that the hydrophilic dendritic branches are localized in this region. TEM images also confirmed the presence of multiple aggregates containing two vesicles (twins for G2-PAMAM-AP and G3-PAMAM-AP) and multi-vesicles (large number of multiple aggregates for G4-PAMAM-AP and G5- PAMAM-AP), due to the increased number of hydrophobic AP units in the outer periphery group of the PAMAM dendrimers. Moreover, SEM and AFM images of G2- and G4-PAMAM-AP supported the self-assembly formation of vesicles of various diameters. The packing parameters of G2–G5-PAMAM-AP were found to be in the range of 0.5–1.0 (as evaluated from EDX O-mapping images), supporting the formation of bilayer vesicular structures. Comparatively larger-sized vesicles were observed under acidic and alkaline conditions, in comparison to that in neutral medium for each generation of amphiphilic dendrimers. At low pH, the generation of ammonium salts from the tertiary amine groups of PAMAM-AP helped to form the polarons from AP units by doping with strong acids, which eventually increased the stability of vesicular aggregates at low pH than that in double distilled water. At high pH, the deprotonation of PAMAM-AP amphiphiles enhanced π–π interactions, leading to the formation of twins or multilayered vesicles. X-ray diffraction (XRD) analyses of the vesicles helped to elucidate the possible molecular packing patterns of G2–G5-PAMAM-AP along with self-assembly driving force analysis. XRD patterns confirmed the presence of a more ordered structure for these higher generation amphiphilic dendrimers, due to the crystallization induced by the result of enhanced π–π interactions (compacted effect) of the strongly emeraldine APs.

#### 3.2.2. Dansyl or 1-(naphthalenyl)-2-phenyldiazene (NPD) shell-modified dendrimer

**Fig. 16:**
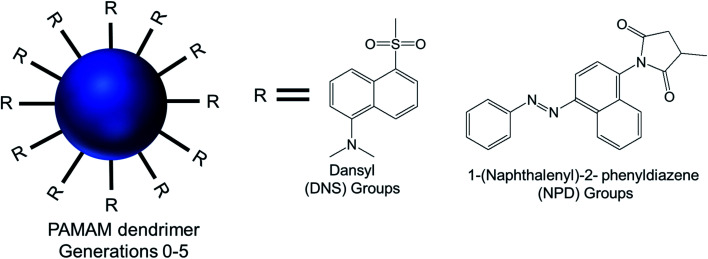
Chemical structure of modified generation 0 to 5 PAMAM dendrimers conjugated to hydrophobic aromatic dansyl (DNS) or 1-(naphthalenyl)-2-phenyldiazene (NPD) groups, *via* sulfonylation and a Michael addition reaction respectively.

Wang and colleagues reported the formation of vesicular assembly from a series of surface modified amphiphilic PAMAM dendrimers, which were synthesized by conjugating various generations (G0–G5) of hydrophilic PAMAM dendrimer with hydrophobic aromatic dansyl (DNS) or 1-(naphthalenyl)-2-phenyldiazene (NPD) groups.^80^ The surface of G0- to G5-PAMAM dendrimers was modified with DNS and NPD groups *via* sulfonylation and a Michael addition reaction respectively to prepare a series of two different set of amphiphiles, namely PAMAM–DNS and PAMAM–NPD (Fig. 16).

AFM and TEM imaging demonstrated the presence of spherical vesicular aggregates of PAMAM–NPD amphiphiles in water, with an average diameter about 100 nm and a bilayer thickness of about 3.2–5.0 nm. DLS study clearly depicted an increase in hydrodynamic radius with increasing generations for PAMAM–NPD-based vesicles (78.4 nm for G0, 57.7 nm for G1, 75.7 nm for G2, and 113.0 nm for G3), whereas no ordered aggregates were obtained for dendrimers with generations higher than G3. Furthermore, PAMAM–DNS also formed vesicular structures with an average diameter of about 135 nm and a bilayer thickness of about 3.2 nm in water. A π–π interaction due to the presence of rigid and short aromatic groups at the periphery of amphiphilic PAMAM dendrimers played an important role, in addition to the amphiphilic nature of modified dendrimers, in the self-assembly process towards vesicle aggregates. This π–π interaction, along with hydrophobic interactions among hydrophobic aromatic moieties conjugated on dendrimer surface, helped these aromatic hydrophobic groups to align in parallel to form the hydrophobic interphase between bilayers to get an energy minimum in water. Upon UV irradiation of NPD groups, G0- to G2-PAMAM–NPD amphiphiles showed the retention of vesicular structures with an increase in aggregate size and a slight decrease in thickness of the vesicle's bilayer due to the photo-isomerization, from the more stable trans form to the less stable *cis* form, of NPD groups leading to a change from a hydrophobic bilayer into a partially interdigitated layer. Based on the measurement of fluorescence intensity of the PAMAM–DNS aggregates in water with increasing dendrimer concentration, the critical aggregation concentration (CAC) values were found to decrease from 6.79 × 10^−5^ to 4.04 × 10^−5^ to 8.79 × 10^−6^ to 5.67 × 10^−7^ M as the generation increased from G0 to G3. This gradual decrease indicated that the aggregate formation was favorable with increasing generation in the order of G3 > G2 > G1 > G0, due to increased π–π interactions in aromatic rings and H-bonding interaction among the dendrimer branches. The aggregation values also decreased from 1.22 × 10^4^ for PAMAM–NPD G0 to 8.79 × 10^2^ for PAMAM– NPD G3, which is a strong indication of decreasing CAC values with increasing generation of dendrimers. Additionally, the calculated critical packing parameter (*P*_c_) values for these aggregates were within the range of 0.5–1.0, supporting the formation of bilayer vesicular aggregates by these amphiphiles in accordance with what observed on TEM images. Cyclic voltammetry, a well-known electroanalytical technique, was used to study the properties of electroactive DNS units attached to PAMAM dendrimer. The cyclic voltammograms of DNS-modified PAMAM dendrimers (G0 to G4) showed a gradual increasing tendency of oxidation potentials with the dendrimer generations (0.77 V for G0- to 0.88 V for G4-dendrimer). This result suggested a gradual decrease in electro-donating ability of PAMAM–DNS with increasing generations, which might be attributed to the more crowded groups at the periphery of the dendrimers at higher generations, as evidenced from a sharp decrease in diffusion coefficients with increasing generation.

#### 3.2.3. Formation of vesicles based on ionic and π–π interactions

**Fig. 17:**
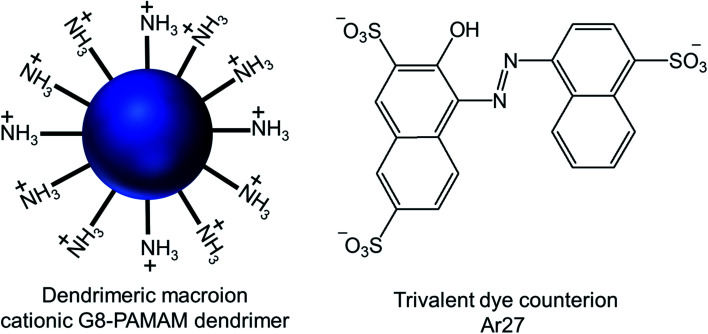
Chemical structure of the cationic generation-8 PAMAM dendrimer and the anionic trivalent sulfonate dye Ar27.

Gröhn and colleagues reported the successful formation of self-assembled supramolecular vesicles due to electrostatic interactions between the cationic G8-poly(amidoamine) dendrimer and the trivalent sulfonate dye Ar27 in aqueous solution, by the so-called counterion condensation (Fig. 17).^81^

Through their investigation based on the results obtained from dynamic light scattering (DLS), static light scattering (SLS), small-angle neutron scattering (SANS), confocal laser scanning microscopy (CLSM) and fluorescence correlation spectroscopy (FCS), they have identified the importance and interplay of electrostatics, π–π interactions and geometric factors between G8-PAMAM dendrimer and Ar27 towards such vesicle formation, even in the absence of any conventional amphiphiles. In order to form vesicles by electrostatic self-assembly, the pH of the solution was kept at such values where amine groups of the dendrimer were positively charged and all sulfonate groups of the dye were negatively charged. DLS study showed the presence of stable aggregates in aqueous solution when using dendrimer excess and a ratio of sulfonate groups to primary amine groups of 1.1 : 1. The hydrodynamic radius of such stable vesicles (*R*_H_ = 130 nm) remained the same after at least 48 h. In presence of a dendrimer excess, small species probably corresponding to non-aggregated excess dendrimer were found to coexist, whereas growth and precipitation were observed in presence of a dye excess. SLS analysis of their nanoscale structure showed a linear behavior in a Zimm plot with a radius of gyration *R*_G_ = 128 nm, indicating the presence of hollow spheres (as the shape sensitive parameter *R*_G_/*R*_H_ = 1), different from solid spheres (*R*_G_/*R*_H_ = 0.775). *R*_G_/*R*_H_ was found to be constant (*R*_G_/*R*_H_ = 1.00 ± 0.05) in spite of the variety of the sizes (*R*_H_) of multiple samples between 110–140 nm. In SANS study, the slope around −2 of the scattering curve (in a log/log representation) at low *q* (after the Guinier regime) confirmed the presence of thin flat structures. Thus such stable aggregates with equal hydrodynamic radius and radius of gyration were identified as the hollow spheres with a thin shell, or vesicles, where all mass points were located on the shell. Moreover, the vesicles formed by interactions between two non-fluorescent dendrimer building blocks and Ar27, successfully served as hosts for a zwitterionic fluorescent dye, FRM 480. This dye did not cause any aggregation with either of the cationic and anionic building blocks because of its zwitterionic nature. The size of these vesicles (*R*_H_ ∼ 140 nm) did not appear to be impacted by such dye encapsulation, as evidenced from DLS study, but the vesicles became visible through CLSM study due to the encapsulation of this fluorescent dye in their microenvironment. Following imaging of dye-loaded vesicles on mica surface, the formation of un-sharp and ‘‘diffuse’’ particles was observed due to the movement of the particles on mica surface as a result of weak immobilization between negatively changed mica surface and positively charged vesicles. Also, the fluorescence correlation spectroscopy (FCS) of the vesicles showed a radius of 1 and 150 nm for only dye and the dye–vesicle system respectively supporting DLS results. Additionally, the vesicles have been shown to be able to encapsulate fluorescently labeled 10 kDa peptide, showing their future potential use in drug delivery.

## 4. Conclusion and perspectives

Polyamine dendrimer-based vesicles, or dendrimersomes, have demonstrated to be highly promising delivery systems of therapeutic genes and drugs for various biomedical applications. This mini-review focused on dendrimersomes prepared from hydrophobically surface-modified hydrophilic PPI and PAMAM dendrimers, as these two dendrimers are currently the most widely used and promising polyamine dendrimers. Additional cationic charges on the surface, stimuli-sensitivity (such as pH, redox, light and temperature) of such dendrimersomes made from various amphiphilic dendrimers helped them to be used as nano-carriers for drugs and genes and to release their cargo in cancer cell-specific environment. The lipid-modified PPI dendrimers were found to form more stable dendrimersomes (with lower CAC) than the drug-modified PPI dendrimers. In addition, cholesterol-modified PPI dendrimersomes showed more efficacy than the fatty acid chain-modified PPI dendrimersomes as drug and gene delivery systems. These lipid-modified, stimuli-responsive polyamine dendrimersomes could be used for drug–gene combination cancer therapy, due to their ability to encapsulate drugs, to form stable complexes with nucleic acids and to enhance overall transfection. They could also be used in future theranostic applications, as a result of their potential entrapment of a therapeutic agent together with an imaging agent.

Although the therapeutic, diagnostic, biosensor efficacies and safety of the dendrimersomes are yet to be confirmed *in vivo*, the early findings of the described studies show that such platforms hold a promising future in cancer management. A further analysis of the structure–property correlation of these newly developed polyamine dendrimersomes should facilitate their development into even more efficacious delivery systems. Overall, because of their ease of preparation, low immunogenicity, embedded stimuli-sensitivities and multifunctional architecture, these polyamine dendrimersomes are likely to be used in therapeutic strategies combining targeting, imaging, diagnostics and therapy. Continued research in this area should therefore enable the preparation of highly specific, highly efficacious dendrimersomes towards the treatment of cancer and other biomedical applications.

## Conflicts of interest

There are no conflicts to declare.

## Supplementary Material
